# The Functions of Major Gut Microbiota in Obesity and Type 2 Diabetes

**DOI:** 10.3390/metabo15030167

**Published:** 2025-03-01

**Authors:** Siman Liu, Zhipeng Tao, Mingyu Qiao, Limin Shi

**Affiliations:** 1 Departments of Nutritional Science, University of Connecticut, Storrs, CT 06269, USA; 2 Department of Nutrition and Food Sciences, Texas Woman’s University, Denton, TX 76204, USA; 3 Departments of Medicine and Biological Chemistry, Johns Hopkins University School of Medicine, Institute for Fundamental Biomedical Research, Johns Hopkins All Children’s Hospital, St. Petersburg, FL 33701, USA

**Keywords:** gut microbiome, *Bacteroidetes*, *Firmicutes*, *Proteobacteria*, *Verrucomicrobia*, clinical trial, obesity, type 2 diabetes

## Abstract

**Background:** Gut microbiomes play a vital role in maintaining whole-body metabolic homeostasis. It has gained significant attention in recent years due to advancements in genome sequencing technologies and a deeper understanding of its relationship with obesity. However, the specific ways in which different microorganisms directly or indirectly influence host obesity, as well as the underlying mechanisms, remain uncertain because of the complexity of gut microbiota composition. **Methods:** In this review, we summarize the roles of the major gut microbiota phyla such as *Bacteroidetes*, *Firmicutes*, *Proteobacteria*, and *Verrucomicrobia* in obesity and type 2 diabetes based on studies published in the past five years on PubMed and Google Scholar. The current therapeutic strategies associated with gut microbiota are also explored from clinical trials, and challenges and future directions are discussed. **Results and Conclusions:** This review will provide a deeper understanding of the functions of major gut microbiota in obesity and type 2 diabetes, which could lead to more individualized and effective treatments for metabolic diseases.

## 1. Introduction

The gut microbiome has gained significant attention in recent years due to a revolution in genome sequencing technologies and improvements in bioinformatic analysis. These developments have enabled researchers to derive novel insights from the vast numbers of human gut microbiome sequences. Particularly, the relationship between the gut microbiome and obesity has been extensively investigated. Employing advanced bioinformatic tools and wet lab methodologies, researchers have not only elucidated correlation but pursued causal relationships. Obesity is known to be influenced by various environmental and genetic factors, including dietary habits, physical activity, cultural and social norms, stress, psychological factors, and heritability coupled with epigenetics. Among these, the gut microbiome has emerged as one of the key causative factors for obesity, predominantly influenced by the types of food and ingredients consumed. Despite the complexity of gut microbiota composition, it remains uncertain how different microorganisms directly or indirectly impact host obesity and the underlying mechanisms. In this review, we summarize the roles of the most abundant and frequently studied microorganisms in obesity and type 2 diabetes based on publications from the past five years on PubMed and Google Scholar. The focus is on major gut microbiota phyla such as *Actinobacteria*, *Bacteroidetes*, *Firmicutes*, *Proteobacteria*, and *Verrucomicrobiota*. The current therapeutic strategies associated with gut microbiota are also explored. Lastly, challenges and future directions are discussed.

## 2. Introduction of Gut Microbiota and Obesity

The gut microbiota is the microbial community throughout the entire gastrointestinal tract, which includes the stomach, small intestine, and large intestine. The gut microbiota consists of a diverse community of microorganisms, including bacteria, viruses, fungi, and other microbes. Chronologically, the gut microbiota is initially established during and after birth and through breast milk prior to weaning, which transfers microbial communities to the infant gut. Once established, the diversity and abundance of gut microbiota remain relatively stable into adulthood. Approximately 40 bacterial species, accounting for 75% of the gut microbiota abundance, are detected in individuals for at least one year. Furthermore, an average 60% of the microbial species residing in the adult gut are retained over five years [1]. Although the human body hosts various microbiotas, the gut contains the most abundant and diverse microbiota compared with other sites such as the skin and vagina. The gut microbiota contains more than 100 trillion microorganisms [2]. The majority of these microorganisms belong to *Firmicutes*, *Bacteroidetes*, *Actinobacteria*, *Proteobacteria*, and *Verrucomicrobiota* [3]; *Firmicutes* and *Bacteroidetes* are predominant families [4]. Varying dominant microbiota inhabit different spaces in the gut. The small intestine, primarily responsible for nutrient absorption, consists of a single layer of absorptive intestinal epithelial cells. The apical surface of intestinal epithelial cells maximizes surface area and is covered by a single, loosely attached mucus layer, with microorganisms residing in its outer layer. Because of the relatively aerobic, acidic conditions and short transit time in the small intestine, facultative anaerobic bacteria predominate here. In contrast, the cecum and colon, offering conditions with fewer antimicrobials, slower transit times, and a lack of simple carbon sources, harbor the most dense and diverse microbial population [5]. Generally, the gut microbiota varies spatially and chronologically throughout every individual. Despite these general patterns, variations in dietary pattern, lifestyle, geography, and cultural background lead to significant differences in gut microbiota composition among individuals.

Obesity is recognized as one of the most concerning diseases worldwide, with individuals exhibiting a body mass index (BMI) over 30 kg/m^2^ classified as obese and those with a BMI exceeding 40 kg/m^2^ classified as class III obese [6]. It is frequently observed that obesity and T2DM co-occur in individuals. Although both conditions are attributed to multiple potential factors, including a lack of exercise, poor diet, and lifestyle habits, the accumulation of lipids in adipose tissues and high-blood-glucose-induced insulin resistance are primary risk factors for T2DM [7,8,9].

## 3. Environmental and Lifestyle Factors Influencing Gut Microbiota Composition

As commonly acknowledged, gut microbiota homeostasis ensures normal function of digestion and nutrient absorption. Additionally, recent studies have discovered that gut microbiota can modulate the immune system [10]; impact human psychology, such as by ameliorating depression via gut–brain bidirectional communication [11,12,13]; and moderate chronic disease such as obesity [14]. Given these significant impacts, it is vital to examine the major factors influencing gut microbiota composition. The age, dietary composition, and socioeconomic and cultural environments of an individual significantly contribute to the diversity of gut microbiota. Despite geographical differences, younger individuals tend to exhibit more diverse gut microbiota within an age range of zero to three years. It is well-acknowledged that the progression of aging post adulthood increases the risk of age-associated disorders through the functional decline of tissues and organs, including the gut. The dynamics of gut microbial populations evolve from birth through the weaning period, where the introduction of solid foods significantly alters the microbiota composition, and stabilize during adulthood, only to gradually change again with advancing age [15]. In the elderly, over 65 years old, reduced intestinal function diminishes nutrient absorption, further altering gut microbiota composition compared with that in the adult period [16].

Although a vast amount of evidence has demonstrated the influence of dietary regimes on gut microbiota, underlying mechanisms remain largely elusive. Recent studies have focused on various aspects of dietary regimes, including eating frequency [17], the division of fasting and feeding cycles [18], and the types and quantities of food and food additives consumed [19].

Different lifestyles such as eating patterns and active practice contribute to different gut microbiota distribution. A randomized controlled trial was performed in overweight men and women who showed metabolic syndrome over one year. The intervention group with energy-restricted Mediterranean diet (MedDiet) and physical activity was compared with a control group with MedDiet. Both resulted in weight loss; however, the intervention group had significantly reduced weight. Significant decreases in *Butyricicoccus*, *Haemophilus*, *Ruminiclostridum 5*, and *Eubacterium hallii* were observed in the intervention group compared with the control group [20]. Significant alterations in the gut microbiota associated with obesity, particularly a reduction in butyrate-producing bacteria, lead to changes in microbial metabolites and components. These changes contribute to metabolic impairments and drive the progression of obesity-related diseases, including insulin resistance and type 2 diabetes [21].

## 4. Current Updates in Gut Microbiota Research

### 4.1. Firmicutes (F) and Bacteroidetes (B)

**Figure 1:**
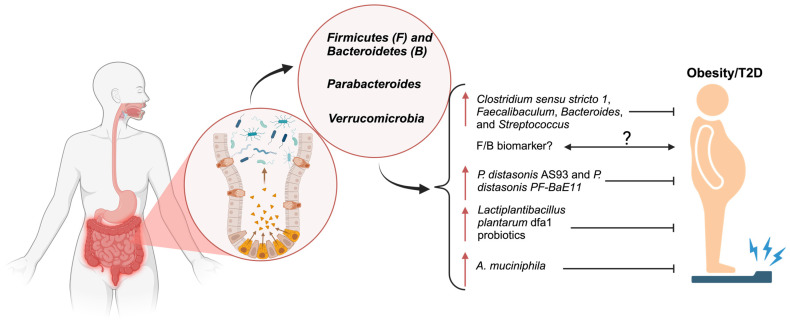
The composition and balance of four major gut microbiota and their roles in obesity. The composition and balance of gut microbiota are pivotal in regulating metabolic processes and influencing the development and progression of obesity. The illustration highlights key bacterial phyla and genera, including *Firmicutes*, *Bacteroidetes*, *Parabacteroides*, and *Verrucomicrobia*, and their roles in obesity and T2D. Imbalance in these microbial communities, characterized by an increased Firmicutes-to-Bacteroidetes ratio and reduced beneficial bacteria such as *Verrucomicrobia*, is associated with heightened energy harvest, metabolic dysregulation, and obesity-related outcomes. ↑, upregulated; ? with ↔, to-be-defined mechanism; diagram created with BioRender.com (accessed on 26 February 2025).

Although there are diverse bacterial species in the gut microbiota, it was recently found that *Firmicutes* (F) and *Bacteroidetes* (B) are the major phyla in the gut microbiota [22,23]. In light of the growing concerns regarding obesity and type 2 diabetes, emerging studies have found that different species, a combination of strains, or the ratio of these two phyla could potentially impact obesity through multiple mechanisms. The ratio of *Firmicutes* to *Bacteroidetes* (F/B) is increasingly considered as a crucial biomarker for obesity regulation and metabolic health. Notably, a higher F/B ratio is associated with an increased likelihood of obesity [22,23]. This biomarker has been extensively evaluated in both animal models (C57BL/6J mice) and clinical trials. For instance, mice treated with collagen peptides alongside a high-fat diet showed a marked reduction in the *Firmicutes*/*Bacteroidetes* ratio. This was accompanied by increases in specific bacterial taxa, including *Clostridium sensu stricto 1*, *Faecalibaculum*, *Bacteroides*, and *Streptococcus*, which are recognized for their antiobesity properties. These changes in gut microbiota also triggered the activation of metabolic pathways, such as polysaccharide degradation and essential amino acid synthesis, that are associated with obesity prevention [24]. Additionally, in a model of high-fat-diet-induced nonalcoholic fatty liver disease (NAFLD) in mice, a higher NAFLD activity score was correlated with altered relative abundances of *Bacteroidetes* and *Firmicutes* [25]. Metagenomic analysis of cecum tissues of New Zealand rabbits showed that *Firmicutes_bacterium_CAG:460* species were significantly higher in high-fat-diet-induced rabbits as compared with those in the control group [26]. Further analysis of gut microbiota from groups of 10 healthy-weight, 10 overweight, and 10 obese individuals, using gene sequencing of the 16S and ITS rDNA regions, revealed a positive correlation between the *Firmicutes*/*Bacteroidetes* (F/B) ratio and BMI. These findings underscore the importance of investigating the F/B community’s role in BMI regulation and metabolic health [27]. In a 24 h dietary recall study of 115 Egyptian women, 82 of whom were obese (59 without metabolic syndrome and 23 with metabolic syndrome) and 33 of whom were of normal weight, fecal microbiota analysis of *Lactobacillus*, *Bifidobacteria*, *Firmicutes*, and *Bacteroidetes* revealed significant findings. Among obese women with metabolic syndrome, the F/B ratio showed positive correlations with total cholesterol and LDL-C and a negative correlation with levels of short-chain fatty acids (SCFAs, which are indirectly produced through microbial fermentation of fibers and play a crucial role in metabolic health and disease regulation) [28,29]. Moreover, a comparison of gastric microbiota composition between obese patients and a control group of normal-weight volunteers diagnosed with functional dyspepsia revealed that obese individuals exhibited significantly lower levels of *Bacteroidetes* and *Fusobacteria*, along with a higher F/B ratio [30] (Figure 1).

The *Firmicutes*-to-*Bacteroidetes* ratio has been observed to increase in individuals with obesity and in various animal models. Over the past decade, this observation has led to the assumption that phylum composition plays a causal role in the rising prevalence of metabolic diseases. However, this statement has been challenged by other studies, which have identified opposing associations between phylum composition and metabolic diseases. In addition, a few contradictory statements have occurred around this F/B ratio. Ducan et al. and Schwietz et al. reported that no significant differences were found between obese and nonobese populations [31,32]. Furthermore, a clinical study analyzing the F/B ratio in stool samples from children at eight time points throughout the first 12 years of life found no relationship between the F/B ratio and BMI z-scores (zBMI). Moreover, the SCFA-producing genera *Subdoligranulum* and *Alistipes* were negatively associated with future BMI in childhood [33]. These findings underscore the need for further, more comprehensive studies with larger sample sizes and more varied sample types to ascertain the validity of the F/B ratio as a biomarker for obesity and type 2 diabetes (T2D).

### 4.2. Parabacteroides

*Parabacteroides*, particularly the species *Parabacteroides distasonis*, has gained attention for its dichotomous role in metabolic health. Wang et al. researched how *P. distasonis* alleviated obesity through the succinate and secondary bile acids produced by *P. distasonis* [34]. In a more recent article, Cuffaro et al. explored the beneficial effects of two *P. distasonis* strains, *P. distasonis* AS93 and *P. distasonis* PF-BaE11. An experiment on HFD-fed C57BL/6JRj male mice demonstrated that supplementation with these two *P. distasonis* strains significantly reduced the body weight of the mice and significantly decreased the expression of all proinflammatory genes [35]. Controversially, it was summarized that *P. distasonis* was widely found to be positively associated with gestational diabetes mellitus (GDM), highlighting the complex role of this phylum in different metabolic contexts [36]. Furthermore, according to a recent study, administration of *Lactiplantibacillus plantarum* dfa1 probiotics in high-fat-induced obese mice for 16 weeks revealed a reduction in the fecal abundance of pathogenic Proteobacteria without an alteration in total Gram-negative bacteria when compared with non-probiotic-treated obese mice [37]. Clinical studies further elucidate the role of the *Parabacteroides genus* in obesity. For instance, prepregnancy obesity has been shown to influence the gut microbiota of both pregnant mothers and their newborns, particularly affecting levels of *Proteobacteria*, which may be implicated in fetal growth. A study examining the effects of prepregnancy obesity on maternal and newborn microbiomes, as well as fetal growth, found that stool samples from mothers who were obese prior to pregnancy exhibited a lower relative abundance of *Proteobacteria* (7.1% vs. 4.1%) compared with those from mothers with normal prepregnancy body weight. Conversely, in the meconium samples, *Proteobacteria* were more abundant in newborns of mothers who were obese before pregnancy (9.0% vs. 8.5%) compared with those of mothers with normal prepregnancy weight [38]. In addition, liver biopsies from the obese group demonstrated lower alpha diversity at the phylum level, as reflected by the Shannon index (0.60 [0.55–0.76] vs. 0.73 [0.62–0.90], *p* = 0.025). Metagenomic profiling further identified a significantly higher proportion of *Proteobacteria* in the obese group (81.0% [73.0–82.4%] vs. 74.3% [68.4–78.4%], *p* = 0.014) [39].

### 4.3. Verrucomicrobiota

*Verrucomicrobiota*, as a phylum of Gram-negative bacteria, contains few described species. Among them, the most abundant bacterium colonizer is *Akkermansia muciniphila (A. muciniphila)*, which is considered as a promising probiotic in the intestinal mucosa of mammals. *Akkermansia muciniphila* has been extensively studied both in animal experiments and clinical studies because of its potential antiobesity effects. It plays a crucial role in regulating lipid metabolism, glucose uptake, and immune response. Specifically, *A. muciniphila* produces short-chain fatty acids, which are essential for host energy metabolism [40]. A study evaluating the preventive effects of both live and pasteurized *Akkermansia muciniphila* MucT (*A. muciniphila*) along its extracellular vesicles (EVs) on HFD-induced obesity demonstrated that body weight, metabolic tissue weight, food consumption, and plasma metabolic parameters were significantly reduced in mice administered *A. muciniphila* [41]. Furthermore, Lin et al. isolated the *Akkermansia municipia* AKK2 strain from the feces of interferon-inducible protein 204^−/−^ (IFI204^−/−^) mice and evaluated its antiobesity effects. Notably, this strain inhibited weight gain and increased the F/B ratio in high-fat-diet (HFD)-fed C57BL/6J mice and beagles [42]. Additionally, supplementation with *A. muciniphila* has been shown to protect mice against high-sucrose-induced glucose intolerance by reducing 3 β-chenodeoxycholic acid (3 β-CDCA) levels while enhancing insulin secretion and fibroblast growth factor 15/19 (FGF15/19) levels [43]. Moreover, in the Geelong Osteoporosis Study cohort, involving 158 men with dual-energy X-ray absorptiometry data, 16S rRNA gene bacterial profiling of stool samples revealed that relative abundance of *A. muciniphila* was inversely associated with high fat mass index (FMI, kg/m^2^) [44]. Similarly, a clinical study involving 182 lean and abdominally obese individuals, with and without newly diagnosed T2D, demonstrated that the abundance of *A. muciniphila* significantly decreased in lean individuals with T2D compared with those without T2D.

Recent advancements in gut microbiota research have unveiled numerous beneficial mechanisms that enhance our understanding of the human body and offer potential solutions for managing obesity and type 2 diabetes. Despite this progress, there remains a substantial scope for further research. With the development of sequencing technologies, the exploration of gut microbiota has deepened significantly. For example, next-generation sequencing technologies have enabled researchers to pinpoint specific target species that could be utilized in microbiota-based therapeutics. Researchers have applied quantitative shotgun metagenomic sequencing to intestinal microbiota from patients and found that a specific strain—*Akkermansia muciniphila*—was positively related to treatment with PD-1 (programmed cell death protein 1, an immune checkpoint receptor expressed on immune cells) [45]. This discovery paves the way for identifying and harnessing beneficial strains that could optimize gut microbiota functions. Moreover, genome sequencing serves as a powerful tool that provides deeper insights into the complex interactions within the gut microbiota and how various treatments can differentially affect this intricate ecosystem [46]. Nevertheless, the inherent complexity of the gut microbiota and the extensive array of interactions between the microbiota and its host demand further advancements in gene sequencing technologies. The urgent need for refined and more capable sequencing tools is evident as we strive to fully decipher the vast potential of gut microbiota in human health and disease.

## 5. Therapeutic Strategies

**Figure 2:**
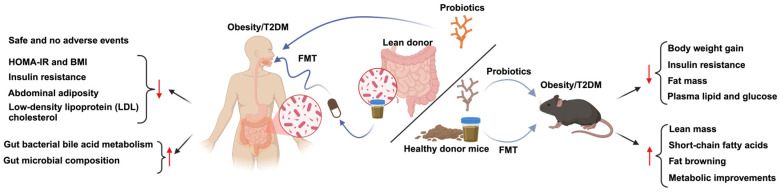
Therapeutic strategies implemented in both mouse models and clinical trials. This schematic diagram illustrates the potential metabolic benefits of fecal microbiota transplantation (FMT) and probiotics in individuals with obesity and type 2 diabetes (T2DM). It depicts the process of gut microbiota transfer from lean donors to both human and mouse models to investigate microbiome-mediated metabolic improvements. ↑, upregulated; ↓, downregulated; diagram created with BioRender.com (accessed on 26 February 2025).

Recent insights into the interactions between obesity and gut microbiota have highlighted the needs for therapeutic strategies. Probiotics as a term was defined in 1974 and later characterized by the Food and Agriculture Organization/World Health Organization in 2020 as “live microorganisms that are beneficial to health if consumed sufficiently” [47]. A recent study reported that *Bifidobacterium lactis YGMCC2013* reduced body weight in diet-induced obese (DIO) mice by altering specific species, further mediating the Il-27 and Tgr5 signaling pathways in adipose tissues [48]. Lee et al. conducted a randomized, double-blind, placebo-controlled, parallel-arm study on the antiobesity effect of *B. lactic* IDCC 4301 among obese women. The results showed that serum triglyceride concentration, total fat, and trunk fat were significantly lowered from probiotics groups. This study suggested that *B. lactis* IDCC 4301 might be related to antiobesity effects in obese women [49]. Prebiotics, composed mainly of inulin-type fructans and galacto-oligosaccharides, have similarly demonstrated efficacy in managing obesity. Li et al. demonstrated that prebiotics such as 2′-fucosyllactose alleviated obesity in HFD mice by increasing energy expenditure in the mice, which further improved the diversity, structure, and composition of the gut microbiota [50]. Combining prebiotics and probiotics, known as synbiotics, has proven more effective than either intervention alone [47,51]. However, different prebiotics and probiotics may be produced differently, and their safety and efficacy might vary as well [52]. A synbiotic including xylooligosaccharide and *Akkermansia muciniphila*, for example, alleviated hyperglycemia and insulin resistance in GDM mice. This synbiotic particularly regulated NKG2D/NKG2DL signaling, which further alleviated insulin resistance [53] (Figure 2). A clinical study on the impact of synbiotic supplementation including *Lactobacillus coagulans SC-208*, *Lactobacillus indicus* HU36, and the prebiotic fructooligosaccharide was conducted among overweight or obese children and adolescents. It was found that the waist–height ratio was decreased significantly after eight weeks [54]. Specifically, this combination improved insulin resistance and protected against obesity-associated inflammation within only 30 days of oral administration [55]. A double-blind randomized clinical trial associated with prediabetes adults showed that synbiotics containing probiotics—*Lactobacillus acidophilus, Bifidobacterium lactis, B. bifidum* and *Bifidobacterium longum*—and inulin were able to decrease the ratio of *Firmicutes* to *Bacteroidetes*, which was beneficial to ameliorating obesity [56]. Recent research has demonstrated that synbiotics may exert effects that are equal to or more beneficial than those provided by probiotics or prebiotics alone. However, the interactions within synbiotic combinations require further exploration. It is necessary to recognize that the functional outcomes of synbiotics cannot be presumed to be additive of the effects of probiotics and prebiotics; rather, these combinations may engage in complex interactions to be further studied [57].

Dietary intervention remains a cornerstone in combating obesity. Intriguingly, increasing dietary fibers even in a high-fat diet has led to beneficial outcomes such as enhanced gut microbiota diversity, increased SCFAs, and elevated energy expenditure compared with a low-fat diet lacking dietary fiber [58]. A study on the effects of whole edible mushroom *Plerutus eryngii* (WPE) intake showed that it inhibited abnormal gain of body weight, which further improved glucose tolerance in HFD-fed mice [59]. Various dietary patterns, such as the Western diet, which is associated with obesity, and the Mediterranean diet, which is rich in antioxidants and fibers and known for its obesity-ameliorating effects, have been studied extensively. Even without reducing the total energy intake, sole modification in dietary behavior was reported to result in healthier body condition, such as lower blood cholesterol [60]. According to a study involving 80 overweight and obese subjects conducted over eight weeks, a high-protein diet increased gut microbiota diversity compared with a diet with normal protein content [61]. More dietary behaviors were shown to alleviate obesity, such as a low-fat vegan diet [62] and a green–Mediterranean diet with increased portions of plant-based foods and reduced meat intake [63].

The recent exploration of fecal microbiota transplantation (FMT) offers a novel approach to obesity management. FMT from lean donors to individuals with obesity has been linked to potential metabolic benefits; however, findings have remained inconsistent across studies (Table 1). Variability in donor microbiota, recipient response, and external factors may contribute to these mixed outcomes, highlighting the need for further research to optimize its therapeutic application. Its impact and the specific practice are still under investigation. Yang et al. found that FMT from methionine-restricted diet (MRD) mice to obese mice promoted fat browning and reduced fat deposition in adipocytes by remodeling gut microbiota [64]. Another study assigned subjects to a Mediterranean diet, green–Mediterranean diet, or normal diet for six months. During months 7 to 14, subjects took capsules containing their own fecal microbiota [65]. Only green–Mediterranean diet intervention accompanied by fecal microbiota transplantation significantly attenuated weight regain [65]. Although fecal microbiota transplantation was proven safe for six months, the sample size in this study was unequal and not large enough, with a majority of male subjects. Controversially, another study focusing on adolescent obesity found that FMT was ineffective in achieving weight loss in subjects aged 14 to 18 years [66], similarly, no significant metabolic effects were found from a clinical study with 24 subjects [67] (Table 1).

## 6. Beyond Gut Microbiota: Gut Microbiota–Brain Axis

The gut microbiota–brain axis has been an emerging central point of recent research because of its intriguing mechanisms through bidirectional communication [75]. This communication involves pathways through which brain function can influence gut health and vice versa, demonstrating a complex interplay [76,77,78,79]. The existence of three bidirectional communication routes has been proved: the neural route, containing the enteric nervous system (ENS) and vagus nerve; the immunological route; and the endocrine route. Each of these plays a distinct yet interconnected role in this axis [80,81]. The brain and the gut are physically linked through the nerve fibers within the vagus nerve and autonomic nervous system (ANS) [80]. The immune system plays a critical role between the brain and the gut microbiota, as the gut hosts the highest number of immune cells among the body organs [82]. Additionally, the endocrine system, as another route between gut microbiota and brain, contributes through regulating the neuroendocrine system of the brain and through the production of hormone-like metabolites by the gut microbiome [83,84], which are associated with various behavioral aspects, including sexual, stress-related, learning, memory, eating, and obesity behaviors [83]. For example, the hypothalamus–pituitary–adrenal (HPA) axis responds to stress-related conditions [85]; cortisol production via the HPA axis can impact the intestinal barrier integrity, subsequently changing the gut environment and then altering the gut microbiota composition [86], leading to a reduction in microbiota diversity [87].

In addition, appetite is closely associated with obesity. The interaction between the central nervous system and the endocrine system impacts the appetite through signals from peripheral organs [80]. The interaction between the central nervous system and the endocrine system is part of the gut microbiota–brain axis.

Furthermore, the decline in cognitive function particularly observed in obese individuals has been associated with changes in gut microbiota [88]. Addressing obesity can thus improve impaired cognitive functions, such as short-term and working memory, by modifying gut microbiota through this axis. Arnoriaga-Rodríguez et al. concluded that specific species such as *Clostridium sp.* 27_14 or *Clostridium* sp. CAG:230 were positively associated with learning and verbal memory, while the certain species within the phylum Bacteroides showed negative association [88]. Considering the complexity of the interactions within the gut microbiota–brain axis, including the brain, appetite, emotions, stress levels, and the immune system, current observational and animal studies have shed light on these mechanisms. However, to validate these findings comprehensively, further studies at the genetic level are essential.

## 7. Challenges and Future Directions

Probiotic and prebiotic interventions are widely used for obesity because of their safety and ease of use [52]. While dietary shifts to healthier alternatives appear safe, adherence challenges may affect study accuracy. The efficacy of FMT and specific microbial strains in obesity reduction require further research. Engineered probiotics show promise in enhancing antiobesity effects [89,90], but clinical studies often face limitations such as small sample sizes [47,67,91] and inconsistent study durations [63,65,91]. Advancing research on diet–gut microbiota interactions could provide critical insights for developing precision nutrition and metabolic disease prevention strategies.

## Figures and Tables

**Table 1:** The efficacy of Fecal Microbiota Transplantation (FMT) in clinical trial for obesity and T2DM.

Clinical Trial	Effects	References
A randomized, placebo-controlled, pilot study in obese patients ([BMI] ≥ 5 kg/m^2^), *n* = 22, follow up on baseline and weeks 1, 4, 6, 8, and 12	- FMT capsules (derived from a lean donor) were well tolerated and resulted in lasting alterations to the intestinal microbiome.	[68]
	- FMT capsules derived from a lean donor were safe but did not lead to reductions in BMI in obese, metabolically healthy patients.	
A randomized, controlled, prospective study in T2DM patients, *n* = 31, follow up on week 4	- FMT alone, as well as FMT combined with metformin, significantly improved clinical indicators such as HOMA-IR and BMI in patients with T2DM.	[69]
	- FMT, with or without metformin, significantly improved insulin resistance, body mass index, and gut microbial composition in patients with T2DM by facilitating the colonization of donor-derived microbiota.	
Dietary-intervention randomized, controlled polyphenols-unprocessed weight-loss trial in abdominally obese or dyslipidemic participants, *n* = 90, follow up in months 6–14	- No adverse events or symptoms associated with FMT were reported.	[65]
	- FMT significantly reduced weight regain in the green–Mediterranean group but had no effect in the dietary guideline or Mediterranean diet groups. Additionally, FMT attenuated waist circumference gain and insulin rebound in the green–Mediterranean group, whereas no such effects were observed in the other dietary groups.	
A double-blind, randomized, placebo-controlled pilot trial in obese, metabolically healthy patients, *n* = 22, follow up on week 4	- FMT enhanced gut bacterial bile acid metabolism and slowed the progression of impaired glucose tolerance compared with the placebo control group.	[70]
	- FMT enriched bacterial species associated with gut bile acid metabolism, including *Desulfovibrio fairfieldensis* and *Clostridium hylemonae*.	
	- *Bacteroides ovatus* and *Phocaeicola dorei* showed positive correlations with unconjugated bile acids, while *Bifidobacterium adolescentis*, *Collinsella aerofaciens*, and *Faecalibacterium prausnitzii* were positively correlated with secondary bile acids.	
A double-blind, randomized, placebo-controlled pilot trial in adults with obesity and mild–moderate insulin resistance, *n* = 24, follow up on weeks 6 and 12	- There were no significant differences in adverse events (AEs), and no serious AEs were associated with FMT.	[67]
	- No clinically significant metabolic effects, including improvements in insulin sensitivity or body composition, were observed during the study.	
A double-blind, randomized, placebo-controlled trial in obese subjects with T2DM, *n* = 61, follow up on weeks 4, 16, and 24	- Repeated FMTs significantly enhanced the engraftment of lean-associated microbiota (*p* < 0.05).	[71]
	- Combining lifestyle intervention with FMT resulted in greater increases in *Bifidobacterium* and *Lactobacillus* compared with FMT alone (*p* < 0.05). It also significantly reduced total and low-density lipoprotein (LDL) cholesterol, as well as liver stiffness, at week 24 compared with baseline (*p* < 0.05).	
A double-blinded, placebo-controlled, multicenter, randomized clinical trial among adult individuals with severe obesity, *n* = 41, 18 months of follow-up	- FMT showed no significant impact on weight loss, either presurgical or postsurgical.	[72]
A randomized, placebo-controlled phase 2 clinical trial in adults with obesity and metabolic syndrome, *n* = 29, follow up on week 6	- Responders to FMT therapy exhibited a higher engraftment rate of specific donor-derived microbes, which were strongly correlated with improvements in insulin sensitivity, as measured by HOMA2-IR.	[73]
A phase II, randomized, single-blind, parallel-arm clinical trial in individuals with type 2 diabetes, *n* = 30, follow up on baseline and at 4 and 12 weeks	- Neither FMT from healthy and lean donors nor a probiotic were effective in improving in weight, body mass index, insulin sensitivity, or HbA1c in patients with T2D.	[74]
A randomized, double-masked, placebo-controlled trial, *n* = 87, follow up on week 26	- FMT had no significant effect on overall weight loss in adolescents with obesity; however, a reduction in abdominal adiposity was observed.	[66]

## Data Availability

No new data were created or analyzed in this study. Data sharing is not applicable to this article.
